# Association of marital status with stage and survival in patients with mycosis fungoides: A population‐based study

**DOI:** 10.1002/cam4.4232

**Published:** 2021-09-04

**Authors:** Ling‐Xiao Xing, Jing Zhang, Hui Shen, Xiao‐Lu Tang, Lu He, Jia‐Zhu Wu, Jian‐Yong Li, Yi Miao

**Affiliations:** ^1^ Department of Hematology The First Affiliated Hospital of Nanjing Medical University Jiangsu Province Hospital Nanjing China; ^2^ Key Laboratory of Hematology Nanjing Medical University Nanjing China

**Keywords:** marital status, mycosis fungoides, prognosis, SEER

## Abstract

**Background:**

Previous studies have shown that marital status was associated with stages and survival in patients with melanoma or Merkel cell carcinoma. To date, the impacts of marital status on stage and survival in patients with mycosis fungoides (MF) have not been determined yet.

**Methods:**

A total of 3375 eligible cases diagnosed from 2004 to 2015 were included from the Surveillance, Epidemiology, and End Results (SEER) database. Association of marital status with stage and survival in patients with MF was analyzed.

**Results:**

Married patients were more likely to be diagnosed at T1 stage (*p *= 0.041). And married patients were less likely to present with lymph node involvement (*p *= 0.007). More favorable overall survival (*p* < 0.001) and cancer‐specific survival (*p* < 0.001) were demonstrated in married patients as compared with divorced patients or widowed patients. A clinically feasible prognostic model including marital status, age, sex, race, and stage at presentation was constructed.

**Conclusion:**

Married marital status was associated with earlier stage at diagnosis and longer survival compared with divorced or widowed marital status in patients with MF.

## Graphical abstract

We found that married marital status was associated with earlier stage at diagnosis and longer survival compared with divorced or widowed marital status. A validated prognostic model that could be potentially used to stratify the patients with mycosis fungoides and guide clinical decision was constructed. Our study highlights the importance of inclusion of marital status as a prognostic factor in mycosis fungoides.

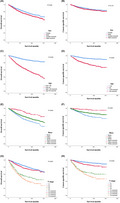

## 1 BACKGROUND

Mycosis fungoides (MF), characterized by a monoclonal proliferation of CD4‐positive T cells, is the most common type of cutaneous T‐cell lymphoma (CTCL) and constitutes almost 50% of all primary cutaneous lymphomas.^1^ According to a newly published study, the incidence of MF increased from 3.0 per million person‐years in the 1970s to 5.9 in the 2010s,^2^ with a higher incidence in Blacks.^3^

MF generally affects adult or elderly patients with a male‐to‐female ratio of 1.6–2.0:1.^1^ Cases of children and adolescents, however, have also been reported.^4^, ^5^ Patients usually present with multiple cutaneous nodules or plaques, and systemic symptoms can be found in 50% of them. Plaques of MF can present as infiltrated, irregular, variably scaling, erythematous, or reddish‐brown lesions. The initial skin lesions have a preference for the extremities and trunk, especially buttocks and other sun‐protected areas. The size of the nodules varies from 5 mm to several centimeters in diameter.^1^ Histopathologically, the most essential characteristic of MF is epidermotropic proliferation of cerebriform lymphocytes forming intraepidermal collections. The infiltration of the lymphocytes is usually within the epidermis in patches and plaques of MF, but the pleomorphic lymphocytes can diffuse through the entire dermis and often to the subcutaneous fat in tumors of MF.^6^ MF has an indolent clinical course lasting over years or decades. Considering distinctive clinicopathologic features, clinical behavior, and/or prognosis, the WHO‐EORTC classification recognizes folliculotropic MF (FMF), pagetoid reticulosis, and granulomatous slack skin as distinct variants of MF compared with classic MF.^1^, ^6^

Currently, MF remains incurable in most patients. Phototherapy, chemotherapy, or radiotherapy are used to treat MF according to the stages and different conditions of the patients.^1^, ^6^, ^7^ The main cause of death of patients diagnosed with MF is systemic involvement or infections. It has been reported that the survival rates are associated with the stages and patients presenting with an early stage at diagnosis have a better prognosis.^2^, ^8^, ^9^, ^10^ Therefore, early diagnosis is essential to improve the prognosis of patients with MF.

The socioeconomic factors, including marital status, income, education level, and others, are found to be associated with the outcomes of cancer patients. Some studies have shown that marriage has a protective effect on patients with malignancies, including Hodgkin's lymphoma, colon, and renal cancer, and others.^11^, ^12^, ^13^, ^14^ The married population is more likely to present with early stage disease^15^ and exhibits better survival outcomes than the unmarried (including single, widowed, separated, and divorced people). For skin cancer including melanoma and Merkel cell carcinoma, it has been shown that being married is associated with early stage and better survival of patients.^16^, ^17^, ^18^ To date, the prognostic impacts of marital status in patients with MF, the most common subtype of cutaneous lymphoma, has not been determined yet.

In this study, we collected data of patients with MF from the Surveillance, Epidemiology, and End Results (SEER) database to assess the effects of marital status on stage at diagnosis and survival outcomes. We found that married patients are diagnosed at an earlier T stage and have more favorable survival outcomes. We determined the prognostic effects of several other parameters. By combining marital status and other prognostic factors, we constructed and validated a prognostic model that could be potentially used to stratify the patients with MF and guide clinical decisions.

## 2 METHODS

### 2.1 Data source

All the data were obtained from the 18 SEER databases sponsored by the National Cancer Institute in the United States. The SEER database collects and publishes cancer incidence, stage, treatment, survival data, and population data such as age, sex, race, and insurance status of 18 population‐based cancer registries, representing about 28% of the US population.^19^

### 2.2 Patient selection and clinical variables

The third edition of the International Classification of Disease for Oncology (ICD‐O‐3) code 9700 was used to identify cases of MF. Patients with MF diagnosed from 2004 to 2015 were included. These cases were chosen as the data regarding AJCC 6th TNM stage. The exclusion criteria were as follows: (1) the marital status was unknown or separated, or having a domestic partner; (2) the AJCC T stage was unknown or not clear (T0 or TX); and (3) survival time was less than 1 month or unknown.

The variables extracted from the SEER database included marital status (married, single, divorced, or widowed), age, race, primary sites, AJCC 6th TNM stage, summary stage, insurance status, and survival months.

### 2.3 Statistical analysis

All the patients with MF were classified into four groups according to the marital status. The categorical variables of each group were presented as frequencies. The differences in categorical variables among these four groups were evaluated using the Chi‐squared test. The Kaplan–Meier method was used to construct the survival curves and the log‐rank test was used to compare the difference. Multivariate analysis was conducted based on the Cox‐regression model. At the end of our analysis, a prognostic model was constructed and validated. All tests were two‐sided and *p *< 0.05 was considered to be statistically significant. All analyses were conducted using SPSS 26.0 statistical software and R software version 4.0.3.

## 3 RESULTS

### 3.1 Basic demographic and tumor characteristics of patients with MF of different marital status in the SEER database

A total of 3375 eligible patients with MF were included in this study. The baseline characteristics of these patients are summarized in Table 1. The median age was 57 years old, with 58.7% being male. There were 2249, 607, 268, and 251 patients in T1, T2, T3, and T4 stages, respectively. Most patients were married (2125, 63.0%). Others were single (767, 22.7%), divorced (242, 7.2%), or widowed (241, 7.1%). The oldest group was widowed, with a median age of 77 years, followed by 60 years for divorced patients, 59 years for married patients, and 44 years for single patients. Male patients constituted a majority in the married group (1350, 63.5%) and were less common in the widowed group (74, 30.7%; *p *< 0.001). A significant difference in the distribution of races was also identified among four groups based on marital status (*p *< 0.001). Since in the United States every patient gets Medicare once they turn 65 years old, we reclassified the insurance status in two age groups and found significantly different distribution in patients with different marital status (Table S1).

**TABLE 1:** Baseline characteristics of patients with mycosis fungoides

	Total	Married	Single	Divorced	Widowed	*p* value
Total, No. (%)	3375	2125 (63.0)	767 (22.7)	242 (7.2)	241 (7.1)	
Age, No. (%)						
≤60	1797 (53.2)	1089 (51.2)	578 (75.4)	114 (47.1)	16 (6.6)	<0.001
>60	1578 (46.8)	1036 (48.8)	189 (24.6)	128 (52.9)	225 (93.4)	
Age, median (IQR), y	57 (46–70)	59 (49–70)	44 (26–60)	60 (52–69)	77 (71–85)	
Male, No. (%)	1980 (58.7)	1350 (63.5)	439 (57.2)	117 (48.3)	74 (30.7)	<0.001
Race, No. (%)						
Black	517 (15.3)	245 (11.5)	178 (23.2)	56 (23.1)	38 (15.8)	<0.001
White	2517 (74.6)	1645 (77.4)	503 (65.6)	177 (73.1)	192 (79.7)	
Other	264 (7.8)	189 (8.9)	59 (7.7)	6 (2.5)	10 (4.1)	
Unknown	77 (2.3)	46 (2.2)	27 (3.5)	3 (1.2)	1 (0.4)	
Site, No. (%)						
Head and/or neck	149 (4.4)	96 (4.5)	28 (3.7)	10 (4.1)	15 (6.2)	0.327
Trunk	704 (20.9)	458 (21.6)	142 (18.5)	52 (21.5)	52 (21.6)	
Extremity	689 (20.4)	439 (20.7)	161 (21.0)	39 (16.1)	50 (20.7)	
Other	1833 (54.3)	1132 (53.3)	436 (56.8)	141 (58.3)	124 (51.5)	
T Stage, No. (%)						
T1	2249 (66.6)	1463 (68.8)	484 (63.1)	157 (64.9)	145 (60.2)	0.041
T2	607 (18)	359 (16.9)	148 (19.3)	48 (19.8)	52 (21.6)	
T3	268 (7.9)	166 (7.8)	63 (8.2)	18 (7.4)	21 (8.7)	
T4	251 (7.4)	137 (6.4)	72 (9.4)	19 (7.9)	23 (9.5)	
N, No. (%)						
N0	2876 (85.2)	1838 (86.5)	641 (83.6)	200 (82.6)	197 (81.7)	0.007
N1	165 (4.9)	81 (3.8)	52 (6.8)	18 (7.4)	14 (5.8)	
N2	22 (0.7)	14 (0.7)	3 (0.4)	4 (1.7)	1 (0.4)	
N3	60 (1.8)	31 (1.5)	20 (2.6)	4 (1.7)	5 (2.1)	
NX	252 (7.5)	161 (7.6)	51 (6.6)	16 (6.6)	24 (10.0)	
M, No. (%)						
M0	3143 (93.1)	1985 (93.4)	710 (92.6)	228 (94.2)	220 (91.3)	0.300
M1	57 (1.7)	28 (1.3)	19 (2.5)	5 (2.1)	5 (2.1)	
MX	175 (5.2)	112 (5.3)	38 (5)	9 (3.7)	16 (6.6)	
Summary stage, No. (%)						
Localized	2765 (81.9)	1766 (83.1)	612 (79.8)	198 (81.8)	189 (78.4)	0.164
Regional	560 (16.6)	335 (15.8)	139 (18.1)	39 (16.1)	47 (19.5)	
Distant	50 (1.5)	24 (1.1)	16 (2.1)	5 (2.1)	5 (2.1)	
Insurance, No. (%)						
Insured or Medicaid	2506 (74.3)	1565 (73.6)	586 (76.4)	171 (70.7)	184 (76.3)	<0.001
Uninsured	43 (1.3)	15 (0.7)	20 (2.6)	8 (3.3)	0 (0.0)	
Other	826 (24.5)	545 (25.6)	161 (21.0)	63 (26.0)	57 (23.7)	

Abbreviations: IQR, interquartile range; y, years.

### 3.2 Association of marital status with stages at presentation

Married patients were more likely to be diagnosed at T1 stage (68.8%) compared with single (63.1%), divorced (64.9%), and widowed (60.2%) (*p *= 0.041). By contrast, widowed patients more commonly presented with T3 and T4 diseases as compared to others. In patients with known lymph node involvement status, married patients (6.0%) were less likely to present with lymph node involvement compared with single (9.8%), divorced (10.8%), and windowed (8.3%) (*p *= 0.007). The frequencies of metastasis did not vary significantly by marital status (*p *= 0.300).

### 3.3 Effects of marital status on survival outcomes of patients with MF

**FIGURE 1:**
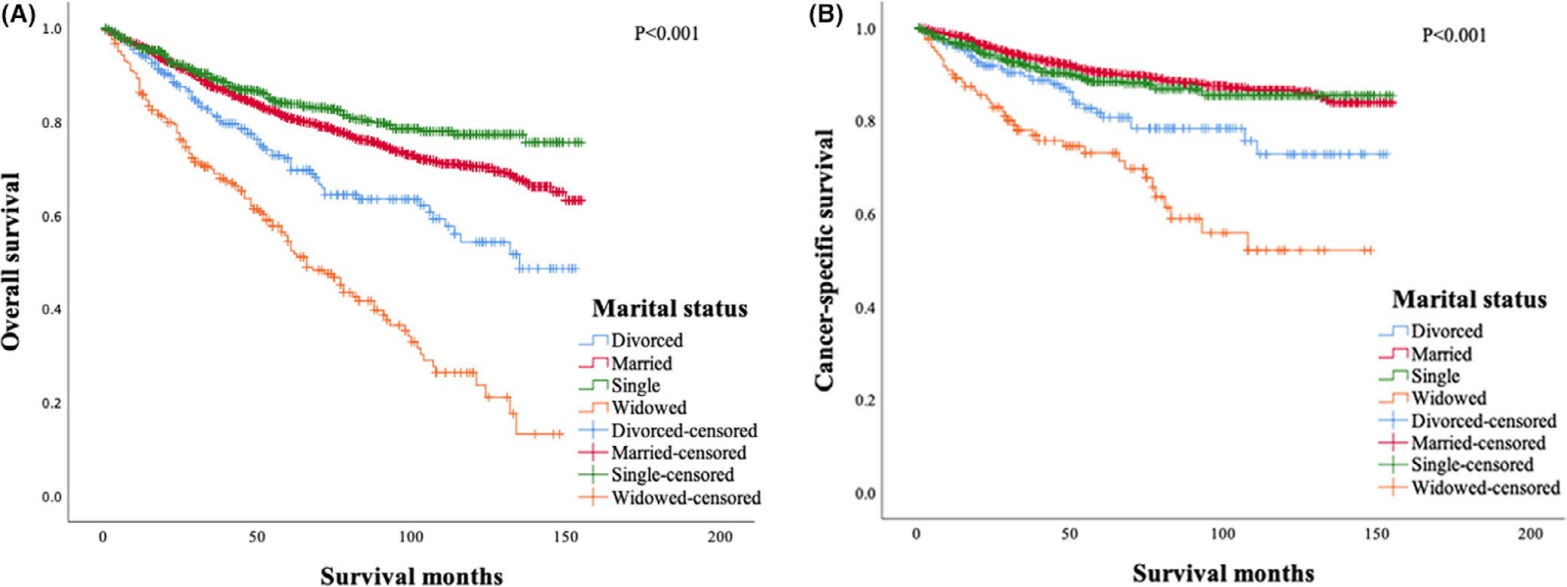
The effect of marital status on the overall survival (A) and cancer‐specific survival (B) of patients with mycosis fungoides

**FIGURE 2:**
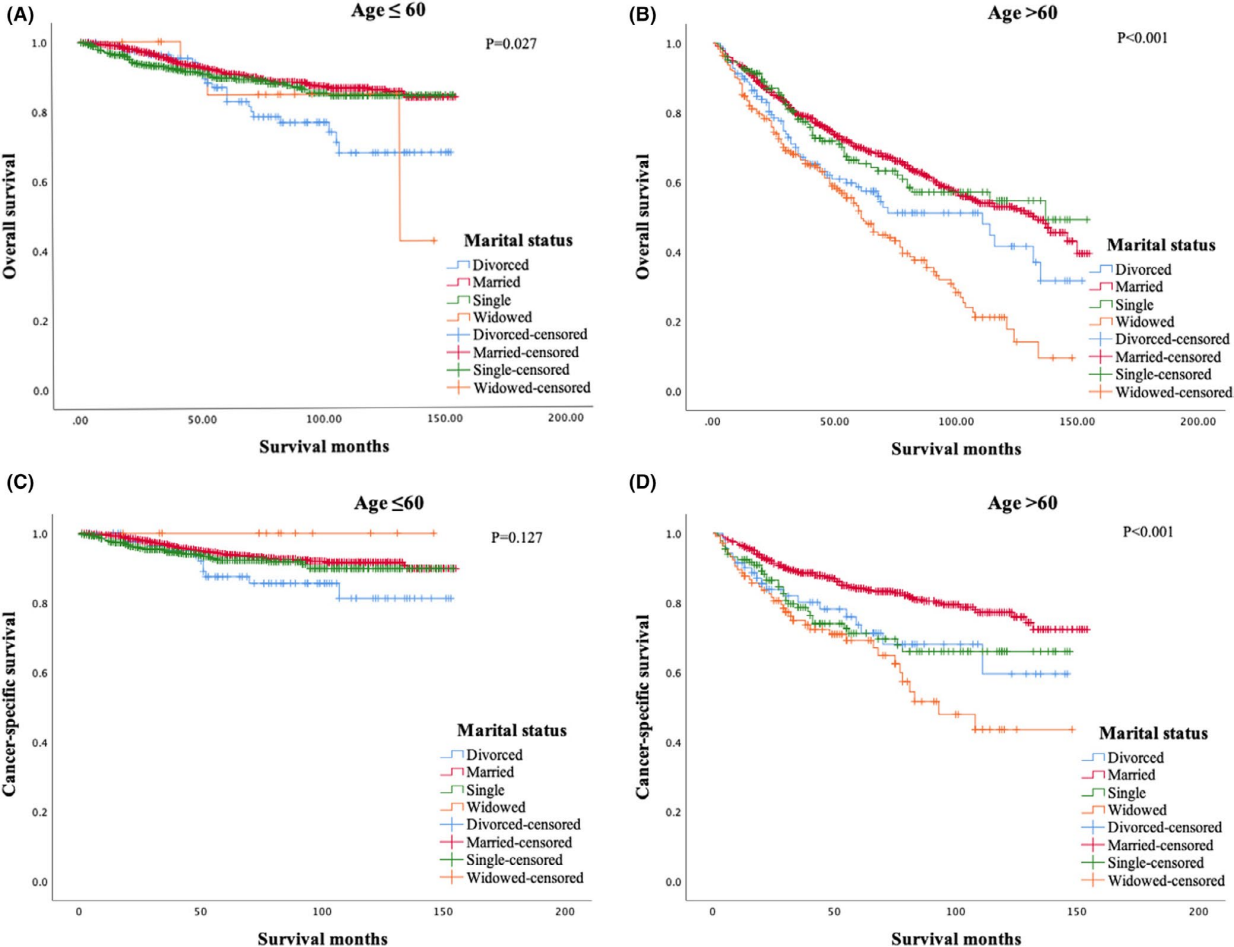
The effect of marital status on the overall survival (A, B) and cancer‐specific survival (C, D) of patients with mycosis fungoides in different subgroups of age

The marital status had a significant impact on the overall survival (OS) (Figure 1A). The 10‐year OS rates were 77.3%, 70.3%, 54.2%, and 26.3% for single, married, divorced, and widowed patients, respectively (*p *< 0.001). We also analyzed the impacts of marital status on the cancer‐specific survival (CSS) in the 2522 patients with CSS data (Figure 1B). The 10‐year CSS rates for single, married, divorced, and widowed patients were 86.5%, 85.4%, 72.8%, and 52.1% (*p* < 0.001), respectively. Married patients showed similar CSS to that of single patients (*p *= 0.316). We then analyzed the effect of marital status in different age subgroups. Married marital status was associated with more favorable OS (*p *= 0.027) (Figure 2A) in patients younger than 60 years old and more favorable OS (*p *< 0.001) (Figure 2B) and CSS (*p *< 0.001) (Figure 2D) in patients older than 60 years old.

It has been reported that from the point of cancer diagnosis, cancer patients have a higher risk of dying from cardiovascular disease (CVD) compared to the general US population and married patients have a lower risk of CVD mortality.^20^, ^21^ Then we chose 2706 eligible patients in our cohort to analyze the effect of marital status on CVD mortality risk in patients with MF and married patients were much less likely to die from CVD than divorced patients and widowed patients (*p *< 0.001) (Figure S1).

### 3.4 The impacts of sex, race, age, and T stage on survival outcomes of patients with MF

**FIGURE 3:**
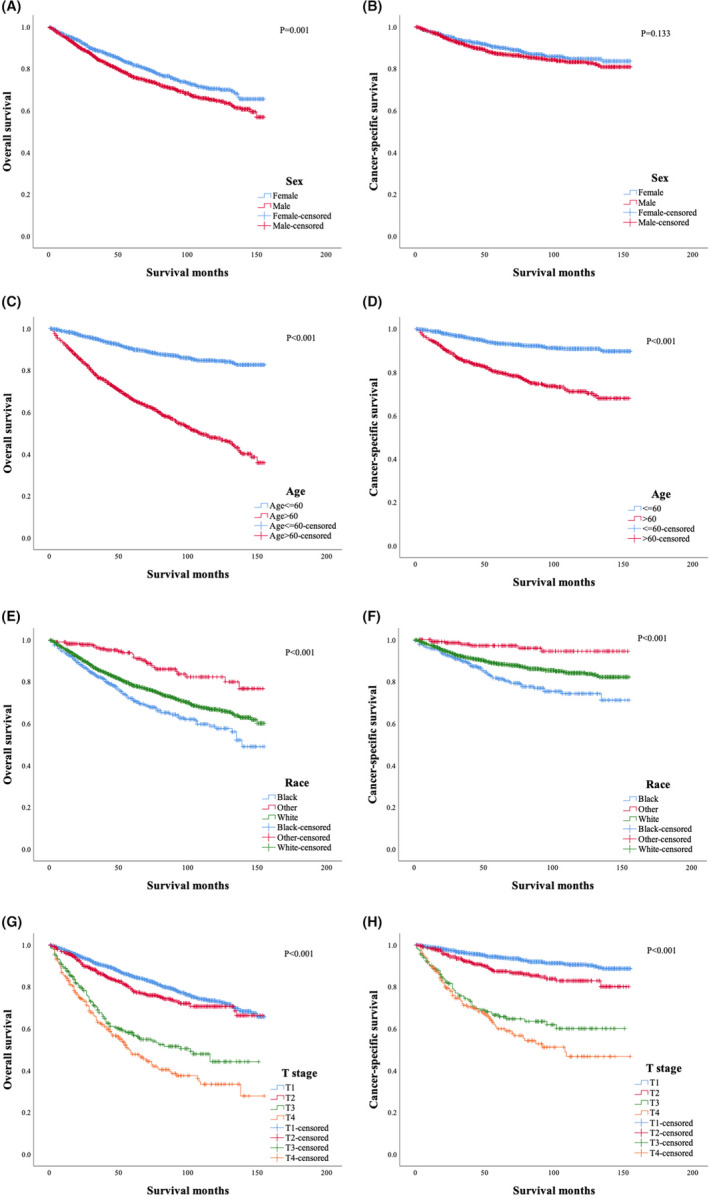
Overall survival and cancer‐specific survival of patients with mycosis fungoides stratified by sex (A, B), age (C, D), race (E, F), and T stage at diagnosis (G, H)

The effects of sex, race, age, and T stage at diagnosis on survival outcomes of patients with MF were also analyzed. Female patients had better OS (Figure 3A) than male patients (*p *= 0.001) while the difference in CSS (Figure 3B) between the two groups was not significant (*p *= 0.133). Patients older than 60 years showed significantly inferior OS (Figure 3C) (*p *< 0.001) and CSS (Figure 3D) (*p *< 0.001) compared to those aged 60 years or younger. The OS (Figure 3E) (*p *< 0.001) and CSS (Figure 3F) (*p *< 0.001) were remarkably different among three racial subgroups, with Black people having the shortest OS and CSS. T stage had significant impacts on survival outcomes. The 10‐years OS rates for patients in T1, T2, T3, and T4 stages were 73.1%, 70.6%, 44.2%, and 33.4% (*p *< 0.001) (Figure 3G), and the 10‐years CSS rates were 90.5%, 82.8%, 60.0%, and 46.7%, respectively (*p *< 0.001) (Figure 3H). However, the pairwise comparisons showed that there was no significant difference in CSS between the T3 stage group and the T4 stage group (*p *= 0.186, result not shown).

### 3.5 Multivariate analysis of prognostic factors for overall survival and cancer‐specific survival for patients with MF

Marital status, age, sex, race, and T stage at diagnosis were identified as independent predictors of OS and CSS by multivariate analysis (Table 2). As compared with married status or divorced status, widowed status independently predicted worse OS (*p *= 0.003) and CSS (*p *= 0.013), however, single status was not significantly associated with worse OS (*p *= 0.735) or CSS (*p *= 0.244). T3 or T4 stage was independently associated with poorer OS (*p *< 0.001 for both T3 and T4 stages) and CSS (*p *< 0.001 for both T3 and T4 stages) as compared to T1 stage. As compared to T1 stage, T2 stage was independently predictive of poorer CSS (*p *= 0.001) but not OS (*p *= 0.066).

**TABLE 2:** Multivariate analysis of prognostic factors for OS and CSS in patients with mycosis fungoides

Variable	Multivariate analysis for OS		Multivariate analysis for CSS		Score
	HR (95% CI)	*p* value	HR (95% CI)	*p* value	
Marital status					
Married	Reference		Reference		
Single	0.965 (0.783–1.189)	0.735	1.192 (0.887–1.603)	0.244	
Divorced	1.444 (1.132–1.843)	0.003	1.650 (1.109–2.455)	0.013	1
Widowed	2.210 (1.793–2.725)	<0.001	2.430 (1.662–3.553)	<0.001	2
Age					
≤60	Reference		Reference		
>60	3.664 (3.076–4.364)	<0.001	3.012 (2.332–3.889)	<0.001	3
Sex					
Female	Reference		Reference		
Male	1.383 (1.186–1.612)	<0.001	1.287 (1.007–1.645)	0.043	1
Race					
Other	Reference		Reference		
White	1.754 (1.198–2.567)	0.004	3.061 (1.439–6.514)	0.004	1
Black	2.734 (1.821–4.104)	<0.001	5.219 (2.392–11.388)	<0.001	2
T stage					
T1	Reference		Reference		
T2	1.211 (0.987–1.486)	0.066	1.727 (1.243–2.399)	0.001	
T3	2.704 (2.193–3.333)	<0.001	5.476 (3.985–7.526)	<0.001	2
T4	3.589 (2.938–4.386)	<0.001	6.201 (4.553–8.445)	<0.001	3

Abbreviations: CI, confidence interval; CSS, cancer‐specific survival; HR, hazard ratio; OS, overall survival.

### 3.6 The development of a new prognostic model

According to the results of multivariate analysis, we developed a prognostic model of MF, which consisted of five variables including marital status, age, sex, race, and T stage at diagnosis. Based on the hazard ratios from the Cox regression analysis for OS (Table 2), every factor with independent prognostic significance was assigned a weighted risk score. Weighted risk scores of one were assigned to divorced marital status, male gender, and White ethnicity; risk scores of two to widowed status, Black ethnicity, and T3 stage at diagnosis; and risk scores of three to age > 60 and T4 stage. The summed final risk scores ranged from 0 to 10 in this cohort. Then these patients were divided into four risk groups based on the summed final risk scores (low‐risk group: 0–2; intermediate‐risk group: 3–5; high‐risk group: 6–7, and very high‐risk group: 8–10).

**FIGURE 4:**
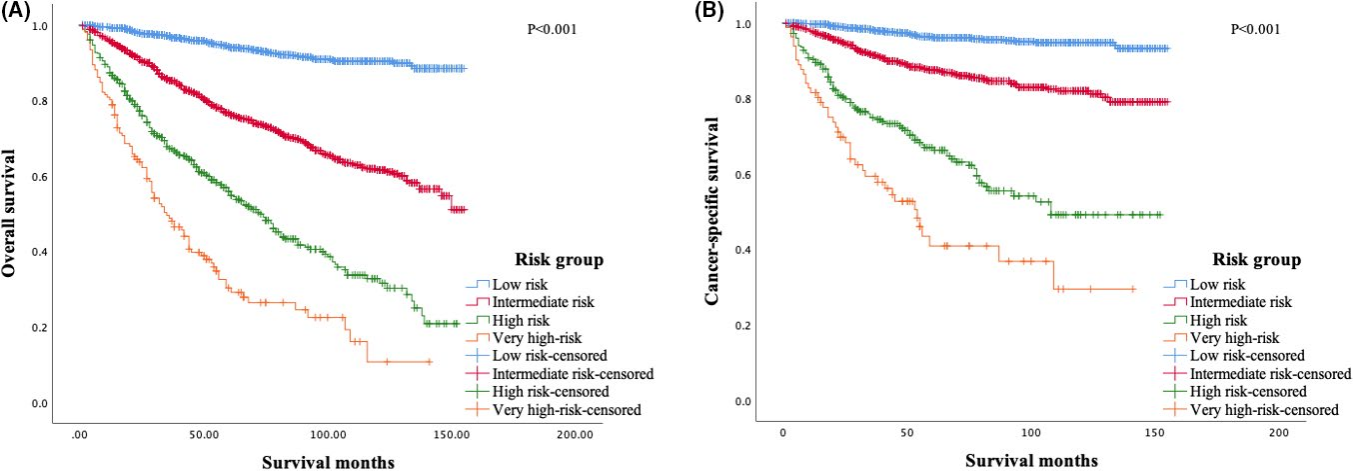
Overall survival (A) and cancer‐specific survival (B) of patients with mycosis fungoides of different risk groups based on the prognostic model

Patients for the four risk groups had remarkably different OS (*p *< 0.001) (Figure 4A) and CSS (*p *< 0.001) (Figure 4B). The 10‐year OS rates for low‐risk, intermediate‐risk, high‐risk, and very high‐risk groups were 90.3%, 61.5%, 32.7%, and 10.7%, respectively. The 10‐year CSS rates for low‐risk, intermediate‐risk, high‐risk, and very high‐risk groups were 94.8%, 82.0%, 49.1%, and 29.4%, respectively.

## 4 DISCUSSION

Mycosis fungoides is the most common subtype of cutaneous T‐cell lymphoma. And the association of marital status with stages and survival outcomes in patients with MF remains to be determined. In this study, we found that married patients tend to have an earlier stage as compared with single, divorced, or windowed patients. And marital status has significant impacts on OS and CSS. Furthermore, after adjustment for T stage, age, and other prognostic factors, marital status was identified as an independent predictor of the survival outcomes of patients with MF, with divorced and widowed statuses independently predicted worse OS and CSS. This study demonstrated that marital status was an important prognostic factor for patients with MF. Moreover, by combining marital status, age, gender, T stage, and race, we developed a risk score that could be used to categorize patients with MF into groups with remarkably different outcomes. This risk score could be potentially used to stratify patients with MF and guide clinical therapeutic options.

We found that married patients had an earlier stage, a finding that was also demonstrated in patients with melanoma or Merkel cell carcinoma. There are several explanations for this phenomenon. For married patients, the skin lesions could be identified by spouse and therefore could be more likely to be diagnosed at an earlier stage. And for unmarried patients, the early stage lesions could be ignored by the patients themselves. Moreover, cancer patients are usually under great depression and anxiety.^22^ Psychological stress may also lead to tumor progression by disturbing normal immune and endocrine system functions.^23^ Married patients could get support and encouragement to see a doctor for examination from their spouses.^24^ And we found married patients had more favorable OS and CSS as compared with divorced or widowed patients. As the clinical stage is an important predictor of CSS and OS, the prognostic role of married status could be partly attributed to its association with the clinical stage. Further multivariate analysis revealed that marital status was an independent predictor of OS and CSS, suggesting marital status could affect the survival outcomes independently of the clinical stage and several other factors. As mentioned above, married patients have more social support, which positively impacts the survival outcomes. Married patients are always assisted by their spouses and are more likely to get timely treatments. We also found that for patients with MF, married patients were at a lower risk of dying from CVD so that may partly explain better prognosis of married patients. In this study, single patients had better OS than married patients and there was no significant difference in CSS between married patients and single patients. Single patients were at a slightly lower risk of dying from CVD as compared with married patients, and this could be attributed to the fact that single patients were much younger than married patients.

Previous studies have identified some prognostic factors for patients with MF. In a cohort study of 1502 patients by Agar et al.,^25^ advanced T stage, the presence of tumor clone without Sézary cells in the peripheral blood, LDH elevation, and the folliculotropic MF subtype were established as independent predictors of poor survival and increased risk of disease progression. In the study by Agar et al., male sex independently predicted poor survival but not increased risk of disease progression. A cutaneous lymphoma international prognostic index (CLIPI) has been developed to predicting survival in patients with MF.^26^ However, further studies demonstrated that this prognostic index could only efficiently stratify early stage patients but not late‐stage patients.^10^, ^27^ In our study, five factors including marital status, age, sex, race, and T stage at diagnosis were established as independent prognostic factors for OS and CSS.

By incorporating these five factors, we constructed a prognostic index, which stratified patients in this cohort into four groups with distinct outcomes. Of these five factors, T stage is available after comprehensive physical examinations. And other four factors are basic demographic factors. Our study established a clinically feasible and robust prognostic tool for risk stratification for patients with MF.

However, our study has several limitations. This is a retrospective study. Some confounding factors may affect the association of marital status with clinical stages. And these confounding factors remain unknown. Besides, other socioeconomic factors, including income levels and education levels, were not included in this study. And classical prognostic factors, including MF pathological subtypes and LDH levels,^28^ were not available in the SEER database. As treatments significantly impact the survival outcomes of MF patients, it is better to include the treatment details when analyzing the prognostic significance of marital status. However, the detailed treatment information was not available in the SEER database.

In summary, our study highlights the importance of inclusion of marital status as a prognostic factor in MF. We demonstrated the association of marital status with the clinical stage in patients with MF. And divorced or widowed marital status was established as independent predictors of worse OS and CSS. And by combining marital status and other prognostic factors, we finally constructed a clinical feasible prognostic index that is robust in predicting the survival outcomes of patients with MF. This research suggests that marital status is a notable issue when doctors are dealing with a patient with MF and enough financial and psychological support should be given to patients without a partner. Further prospective studies, which may include more prognostic factors and treatment details, are needed to more precisely elaborate on the impacts of marital status on the clinical stage and prognosis of patients with MF.

## CONFLICT OF INTEREST

The authors declare no competing interests.

## ETHICS APPROVAL AND CONSENT TO PARTICIPATE

A Research Data Agreement Form was required by the National Cancer Institute's SEER Program prior to access to the de‐identified SEER dataset. Since the de‐identified data were used, approval from the Ethics Committee of the First Affiliated Hospital of Nanjing Medical University and patients’ informed consent were not required.

## Supporting information

Supplementary Material

## Data Availability

All data used in this paper are accessible from Surveillance, Epidemiology, and End Results (SEER) database after a reasonable submission of a request for access to the data at https://seer.cancer.gov/.
