# Supplementary material for: Association of marital status with stage and survival in patients with mycosis fungoides: A population‐based study
Source: Cancer Med. 2021 Sep 4;10(20):7320–9. doi: 10.1002/cam4.4232 (PMC8525132; doi:10.1002/cam4.4232)

Supplemental table 1. Insurance status in different age groups

| **Age<65 years old** | **Total** | **Married** | **Single** | **Divorced** | **Widowed** | **P value** |
| --- | --- | --- | --- | --- | --- | --- |
| Insurance status(%) |  |  |  |  |  |  |
| Insured or Medicaid | 1567(74) | 949(73.5) | 497(76.6) | 102(68) | 19(70.4) |  |
| Uninsured | 42(2) | 15(1.2) | 20(3.1) | 7(4.7) | 0(0) | P=0.002 |
| Other | 41(27.3) | 327(25.3) | 132(20.3) | 41(27.3) | 8(29.6) |  |
| **Age≥65 years old** | **Total** | **Married** | **Single** | **Divorced** | **Widowed** |  |
| Insurance status(%) |  |  |  |  |  |  |
| Insured or Medicaid | 1567(74) | 949(73.5) | 497(76.6) | 102(68) | 19(70.4) |  |
| Uninsured | 42(2) | 15(1.2) | 20(3.1) | 7(4.7) | 0(0) | P=0.033 |
| Other | 41(27.3) | 327(25.3) | 132(20.3) | 41(27.3) | 8(29.6) |  |

Supplemental figure 1. The effect of marital status on the cardiovascular disease mortality risk in patients with mycosis fungoides


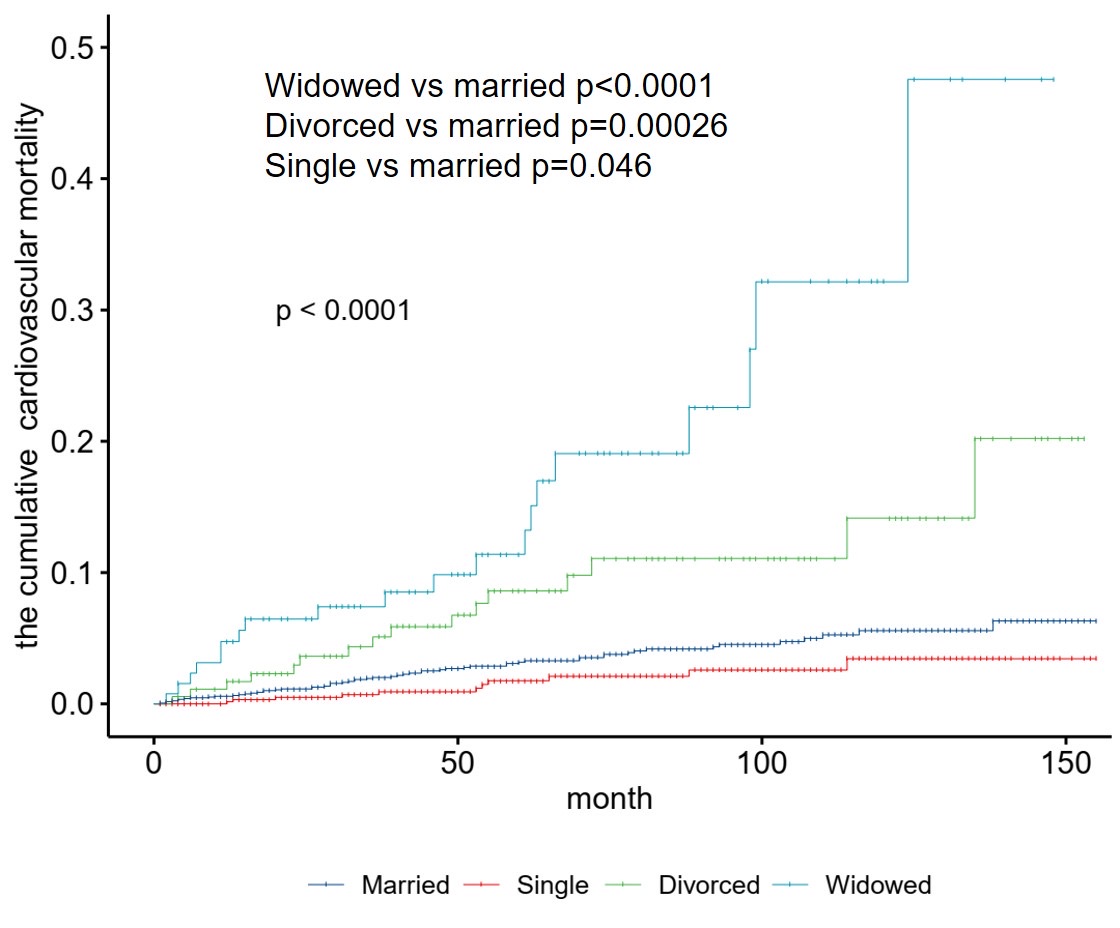

Supplement: Supplementary file 1 — Supplementary Material [file CAM4-10-7320-s001.docx]
